# Behavioral Indicators and Verbal Judgments in the Perception of Translation Quality: A Cognitive Experimental Study

**DOI:** 10.3390/bs16050747

**Published:** 2026-05-11

**Authors:** Xin Huang, Xiang Zhang

**Affiliations:** 1 School of Translation Studies, Jinan University, Zhuhai 519070, China; huangxin@jnu.edu.cn; 2 Faculty of Languages and Translation, Macao Polytechnic University, Macao 999078, China

**Keywords:** translation evaluation, response time, verbal reports, phrase order, semantic expression

## Abstract

This study investigates the cognitive dynamics of translation-related evaluation by comparing online behavioral responses with retrospective verbal reports. Using a 2 × 2 within-subject design, 44 Chinese–English bilingual participants evaluated English translations of Chinese haiku-like texts in a judgment task. Two factors were manipulated: phrase order (congruent vs. incongruent) and local semantic expression type (literal vs. non-literal). Response times (RTs), rating scores, and post-task interview data were collected. The results showed a divergence across indicators. Phrase order significantly affected both RTs and rating scores, with congruent structures associated with faster responses and higher ratings. However, most participants did not report phrase order as a relevant factor in their verbal explanations. In contrast, semantic expression type influenced RTs but did not produce reliable differences in rating scores. Importantly, a significant interaction between phrase order and semantic expression type was observed in rating data but not in RTs. This pattern suggests that sensitivity to local semantic variation may depend on structural processing conditions during evaluative judgment. These findings indicate that behavioral indicators and verbal judgments capture different aspects of translation-related processing and evaluation. While RTs provide a sensitive index of processing effort, rating scores reflect more global evaluative outcomes, and verbal reports capture participants’ retrospective interpretations. These findings are discussed within the framework of dual-system theory, demonstrating how implicit cognitive processing (primarily indexed by response times) and explicit conscious reflections offer complementary insights into the perception of translation quality. The findings also highlight the value of combining behavioral and metacognitive methods in studying translation evaluation.

## 1. Introduction

Translation quality has long been discussed within cross-cultural communication and translation studies, where evaluative judgments are often influenced by linguistic, functional, and interpretive perspectives (Afrouz & Parsa, 2023; House, 2019). As the final stage of the translation process, evaluation plays a crucial role in determining the pragmatic or dynamic equivalence of the target text. Numerous scholars have proposed frameworks and approaches from linguistic and functional perspectives, such as those developed by Williams (2004), House (1997, 2001, 2004, 2014), and Reiss and Vermeer (1984). Traditional approaches, including equivalence-based criteria (e.g., stylistic fidelity), rely heavily on experts’ introspective judgments, which may be influenced by individual interpretation and may lack empirical validation (Newmark, 1988; Nida, 1964). While cognitive science has made substantial progress in understanding bilingual language processing since the 1990s (Green, 1998; Kroll & Stewart, 1994), translation studies have only partially incorporated experimental approaches capable of examining processing-related phenomena.

Cognitive theories suggest that the translation process involves both conscious strategy selection and more automatic processing mechanisms (Shreve & Angelone, 2010). A similar distinction may also be relevant to translation quality evaluation, which can be understood as a process involving both immediate perceptual responses and more reflective judgments. Cognitive methods such as think-aloud protocols and behavioral techniques have contributed to the study of translation processes. However, how different types of measures relate to each other in capturing translation-related processing remains insufficiently understood. In particular, it is not yet clear to what extent behavioral measures and verbal reports reflect similar or different aspects of translation evaluation.

A key concern in translation activity is how source-text meaning is represented and interpreted in the target language. In semantics, the principle of compositionality—often traced back to the work of Frege (1948), originally published in 1892—proposes that the meaning of an expression is derived from the meanings of its parts and their syntactic arrangement. Although this principle has been debated due to its simplified representation of meaning construction, it remains useful as an analytical heuristic for examining how structural and lexical factors may influence interpretation. In line with this perspective, the study operationalizes two dimensions: (1) the arrangement of components (phrase order), and (2) the realization of meaning within components (semantic expression), that are examined in relation to translation processing and evaluation.

Phrase order refers to the arrangement of sentence components such as the subject, predicate, and complement. It may influence how information is structured and how alignment between source and target sentences is perceived. It should be noted that phrase order is not treated as an indicator of translation quality per se, but as a factor that may affect processing fluency and the thematic structure of a sentence. A number of theorists have emphasized the role of structural reorganization in translation (Catford, 1965; Vinay & Darbelnet, 1995).

Semantic expression within components refers to variation in how meaning is realized within components of a sentence. Such variation may occur at the lexical level (e.g., differences in word choice) or at the phrasal level (e.g., alternative formulations). While some aspects of lexical processing may be relatively automatic, variation in semantic expression may still introduce subtle differences in processing effort and evaluative judgment. Prior research has examined these factors individually through text-based analyses (Balla & Siddiek, 2017; Ibrohimovna, 2025; Istoda, 2026) or retrospective interviews (Bernardini, 2001). However, relatively limited research has examined how these factors are reflected across different types of measures within a single experimental framework.

This study therefore investigates how behavioral indicators (e.g., response times and rating scores) and verbal reports relate to each other in reflecting processing differences during translation evaluation. Using poetry translation as a case that requires sensitivity to both structural and semantic variation, the study explores whether different types of measures may provide complementary perspectives on translation-related processing, rather than a single unified index of quality or performance.

To situate this investigation, the following section reviews relevant theoretical and methodological perspectives on translation processing and evaluation.

## 2. Literature Review

This review contextualizes the study within dual-system theories of cognition (Section 2.1), contrasts verbal and behavioral approaches in translation (Section 2.2) to identify current methodological gaps (Section 2.3), and establishes the need for an integrated paradigm (Section 2.4).

### 2.1. Dual-System Theories and Processing Distinctions

Dual-system theories distinguish between fast, automatic processing (System 1) and slower, more deliberate processing (System 2) (Evans, 2003; Frankish, 2010; Kahneman, 2013). This distinction has been widely applied in studies of language use, where it is often associated with the contrast between relatively automatic linguistic operations, such as syntactic parsing and lexical retrieval, and more controlled processes involving metalinguistic awareness and strategic decision-making (Clahsen & Felser, 2006; Ellis & Roever, 2018; Paradis, 2004, 2009; Ullman, 2001).

In bilingual contexts, language processing may involve the interaction of these two modes, depending on factors such as task demands, proficiency, and contextual complexity (Mishra & Prasad, 2024; Wu & Thierry, 2010). Translation, as a complex bilingual activity, is therefore likely to engage both more automatic and more controlled forms of processing (Dong & Li, 2020; Togato et al., 2016, 2025; Wang & Chen, 2013). Previous research suggests that routine or structurally straightforward cases may rely more on automatic processing, whereas more complex or less conventional cases may require increased involvement of controlled processing (Carl, 2013; Deckert, 2017).

This distinction provides a general framework for considering variability in translation-related processing, without presupposing a strict separation between the two systems. Rather, the two modes are often understood as interacting and dynamically shifting during language use (Carl, 2025; Deckert, 2016, 2017).

### 2.2. Methods in Translation Research: Verbal and Behavioral Approaches

Research on translation processes has employed a range of methodological approaches to investigate how translation is performed and evaluated. Among these, verbal-report methods and behavioral measures represent two widely used but methodologically distinct approaches.

Verbal-report methods, most notably think-aloud protocols (TAPs), have been extensively used to capture translators’ consciously accessible reasoning during task performance. By eliciting concurrent or retrospective verbalizations, these methods provide insights into decision-making processes, problem identification, and strategy use (Bernardini, 2001; Eftekhary & Aminizadeh, 2012; Indarti, 2024; Jääskeläinen, 2002; Kussmaul & Tirkkonen-Condit, 1995). As such, they are particularly suited to examining reflective and metalinguistic aspects of translation activity (Dejica & Grigoraș, 2025; Kussmaul & Tirkkonen-Condit, 1995; Sun, 2011).

In contrast, behavioral approaches focus on observable performance indicators that can be recorded during task execution. These include measures such as response times, eye movements, and keystroke patterns, which have been used to investigate the temporal dynamics and processing characteristics of translation (Alves et al., 2009, 2011; Carl, 2025; Francis & Gallard, 2005; Jakobsen, 2011; Rojo López & Ramos Caro, 2014; Schaeffer et al., 2019; Su & Li, 2019; Torres et al., 2025). These measures provide access to the temporal dynamics of processing and are often interpreted as indexing aspects of cognitive activity that unfold during task performance.

In addition to temporal and process-oriented indicators, rating scores are also frequently used in translation research as a form of evaluative response. Typically implemented as Likert-type scales, ratings capture participants’ immediate judgments of translation quality without requiring extended verbalization (Han, 2020; Han et al., 2024, 2025). While they involve an element of conscious evaluation, they are typically less elaborated than verbal reports and may therefore reflect more immediate evaluative responses (Han et al., 2025).

Taken together, these approaches provide complementary perspectives on translation activity: verbal reports offer access to articulated reasoning, whereas behavioral measures and rating responses capture different aspects of performance as it unfolds during processing.

### 2.3. Methodological Gap: Relating Behavioral Indicators and Verbal Judgments

While verbal-report and behavioral approaches have both contributed to the study of translation processes, their relationship remains insufficiently clarified. In cognitive research more broadly, studies have shown that observable behavioral responses and consciously accessible reports do not always align, particularly in tasks involving automatic or time-sensitive processing (Carrasco & Spering, 2024; Micher et al., 2024). In bilingual language use, for example, phenomena such as implicit language activation and lexical competition have been shown to affect performance even when they are not reflected in self-reported awareness (Kim & Kim, 2018; Thierry & Wu, 2007; Villameriel et al., 2022).

In the context of translation, similar observations have been reported. Process-oriented studies suggest that aspects of translation behavior captured through behavioral measures, such as eye movements or temporal patterns, may not always correspond directly to explicitly articulated judgments or strategies (Jakobsen, 2014; O’Brien, 2011). At the same time, verbal reports remain an important source of data on reflective decision-making and evaluative reasoning.

Despite these developments, relatively few studies have examined how behavioral indicators and verbal judgments relate to each other within a single research design, particularly in the context of translation evaluation. As a result, it remains unclear to what extent these different types of data converge, diverge, or provide complementary insights into translation-related processing. This lack of systematic comparison constitutes a methodological gap that the present study seeks to address.

### 2.4. Methodological Integration in Translation Research: Implications for the Present Study

The incorporation of psycholinguistic and cognitive methods into translation research has led to increasing methodological diversification, driven by a growing interest in capturing translation as a cognitively grounded and temporally unfolding activity (Hansen-Schirra & Nitzke, 2020; Yuhan et al., 2024). Building on established behavioral and process-oriented approaches, recent studies have expanded the methodological repertoire to include neurophysiological techniques such as event-related potentials (ERP), electroencephalography (EEG), and functional near-infrared spectroscopy (fNIRS) (Christoffels et al., 2013; García et al., 2016; He & Hu, 2022).

Within this expanding methodological landscape, integration remains uneven and domain-specific. Triangulation is relatively common within behavioral and process-oriented approaches—such as combinations of eye-tracking, keystroke logging, and corpus-based data—yet is largely confined to this domain. By contrast, cross-domain integration is more limited: neurophysiological approaches tend to be implemented within focused and methodologically self-contained designs, and even within behavioral paradigms, certain combinations (e.g., response-time measures and verbal-report techniques) are still relatively rare.

As a result, it remains unclear how behavioral indicators of processing effort (e.g., response times), evaluative responses (e.g., rating scores), and retrospective verbalizations are related, and whether they capture overlapping or distinct dimensions of translation-related cognition. This issue is particularly salient in evaluative contexts, where judgments are made under time constraints but later reconstructed through reflective reasoning.

The present study addresses this gap by examining the relationship between behavioral indicators (response times and rating scores) and verbal reports within a single experimental framework. Rather than treating these approaches as independent or competing sources of evidence, the study investigates the extent to which they provide complementary perspectives on translation processing and evaluation, thereby contributing to a more integrated account of translation-related cognition.

Beyond its implications for translation process research, the integration of cognitive and behavioral methods also holds significant potential for translation teaching and assessment. In pedagogical contexts, measures such as response times, keystroke patterns, and verbal reports can offer fine-grained insights into learners’ processing difficulties, strategic behavior, and metacognitive awareness, thereby supporting evidence-based feedback and instructional design. In assessment contexts, such data enable a shift from purely product-oriented evaluation toward a more process-informed understanding of translation competence and performance variability.

## 3. Research Questions

This study aims to investigate how behavioral measures (response times and rating scores) and verbal reports differ in reflecting the effects of linguistic manipulations during translation evaluation. It addresses the following three research questions:

1. How do response times (RTs) and rating scores differ from verbal reports in reflecting the effects of phrase order manipulation during translation evaluation?
2. To what extent does local variation in semantic expression modulate processing speed (RTs) and rating scores during translation-related processing and evaluation?
3. To what extent do behavioral measures and verbal reports show convergent or divergent patterns in translation-related processing and evaluative behavior?

By situating translation studies within the paradigms of cognitive science, this research not only contributes to refining models of bilingual processing but also offers methodological insights for experimental design in interdisciplinary language research.

## 4. Methodology

This section outlines the methodological framework of the study. It details the experimental design (Section 4.1), presents participant demographics (Section 4.2), explains the construction of the bilingual haiku materials (Section 4.3), and describes the sequential procedures for both the behavioral and verbal report tasks (Section 4.4), followed by the statistical analysis plan (Section 4.5).

### 4.1. Experimental Design

The study examines how two linguistic factors (phrase order and semantic expression type) influence translation evaluation across two complementary methodological components: a behavioral task and a verbal report task.

For the behavioral component, a 2 × 2 within-subject factorial design was employed, with phrase order (congruent vs. incongruent) and semantic expression type (literal vs. non-literal) as independent variables. The semantic expression manipulation was restricted to localized variation within individual components (i.e., within a single line), while overall meaning was preserved. Dependent variables included explicit ratings of translation quality and response times (RTs).

The dependent variables included:

1. Explicit Ratings: Translation quality scored on a 5-star scale (1 = poor, 5 = excellent). Ratings reflecting participants’ overall impression of translation quality.
2. Implicit Metrics: Response times (RTs) recorded from stimulus onset to button press.

In addition, a verbal report component was included to capture participants’ explicit awareness of the experimental manipulations. This component did not follow a factorial structure but instead involved post-task interviews, in which participants were asked to reflect on the factors influencing their judgments. Responses were subsequently coded and analyzed separately.

### 4.2. Participants

Forty-four Chinese-English bilinguals (15 males, 29 females; mean age = 22.6 years) were recruited from the University of Macau, including 26 undergraduates and 18 postgraduates. All participants were native Mandarin speakers with no prior expertise in translation or haiku poetry. Their English proficiency was rigorously controlled: undergraduates had passed standardized exams (e.g., Chinese National College Entrance Examination), while postgraduates met higher thresholds (e.g., TOEFL iBT ≥ 80 or IELTS ≥ 6.0). Participants were unbalanced bilinguals, having acquired English as a second language after age 8. Written informed consent was obtained, and the study protocol was approved by the university’s ethics committee.

### 4.3. Materials

Ten Chinese-English haiku pairs were selected from *Lighting the Bridge to the Moon: One Hundred and Eleven Macao Haiku* (Kelen, 2012), a collection of collaboratively produced bilingual poems created by students under guided instruction. The poems are characterized by relatively simple and accessible language, with minimal reliance on culturally specific imagery or complex metaphorical interpretation. They are not widely known canonical works, which helps reduce the influence of prior familiarity and culturally specific interpretation.

Importantly, the Chinese and English versions were developed in parallel rather than through a strict source–target translation process. As a result, the two versions can be considered closely aligned bilingual expressions of the same underlying content. For the purposes of the present study, these aligned pairs are treated as functionally equivalent to source–target pairs, providing a controlled basis for examining how variations in structural arrangement and semantic expression influence evaluation.

The haiku format provides a clear formal segmentation of linguistic components. Each line typically corresponds to a relatively self-contained syntactic or semantic unit (e.g., subject, predicate, or modifier). As a result, phrase boundaries are explicitly marked, allowing controlled manipulation of structural arrangement without introducing ambiguity in constituent structure.

Given these properties, the Chinese version of each poem was treated as the manipulable input, while the English version served as a stable reference for evaluation. For each item, four Chinese variants were systematically constructed by manipulating two factors:

(1) Phrase Order:
  - Congruent—the sequence of lines corresponds to the structure reflected in the English text;
  - Incongruent—the order of lines is rearranged while preserving overall interpretability.
(2) Local Variation in Semantic Expression:
  - Literal—the expression closely matches the formulation reflected in the Chinse text;
  - Non-literal—the expression deviates from the target formulation, either through lexical substitution or alternative phrasing, while preserving overall meaning.

It should be noted that phrase order is not treated as an indicator of translation quality per se, but as a factor that may influence processing fluency and perceived alignment between the two texts. Likewise, the manipulation of local variation in semantic expression captures variation in how meaning is realized within components, which may occur at the lexical or phrasal level. This manipulation was implemented at the level of individual lines, resulting in localized variation that did not alter the overall meaning of the poem.

The distinction between “literal” and “non-literal” expression is used as an operational simplification to group different types of variation under a unified condition.

Each manipulated Chinese variant was paired with the same English text, resulting in four experimental conditions per item. This design allows for controlled examination of how variations in structural arrangement and semantic expression influence participants’ evaluation, while keeping the reference text constant across conditions. Representative examples of the four conditions are provided in Table 1.

Ten filler items containing deliberate grammatical anomalies were included to reduce participants’ awareness of the experimental manipulations.

All materials were pretested in a pilot study (N = 30 bilinguals), which confirmed that the manipulated conditions produced distinguishable evaluative responses without substantially altering overall comprehensibility.

Although the number of items was relatively limited, supplementary item-level inspections suggested that the observed effects were consistent across stimuli and were not driven by a small subset of items. Nevertheless, future studies using larger and more diverse stimulus sets, as well as mixed-effects modeling approaches, would further strengthen the generalizability of the findings.

### 4.4. Procedure

The experiment consisted of two phases: a behavioral task and a verbal report session.

**Figure 1:**
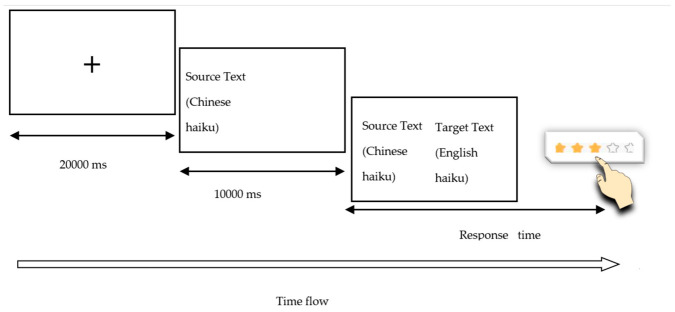
Schematic illustration of the behavioral task procedure.

During the behavioral task, participants were seated approximately 60 cm away from a 23-inch Tobii TX300 eye tracker (Tobii Technology AB, Danderyd, Sweden) (sampling rate: 300 Hz) and a response keyboard. Each trial began with the presentation of a fixation cross for 2000 ms, followed by a Chinese haiku displayed on the left side of the screen. After a 10,000-ms delay, its English translation appeared on the right side. Although an eye-tracking device was used during data collection, the present study focuses on response times and rating data only. Participants were instructed to evaluate the overall quality of the translation based on their immediate judgment, without specific criteria provided, in order to capture intuitive evaluative responses. Ratings were given on a 5-point Likert-type scale (1 = very poor, 5 = very good), reflecting participants’ overall impression of translation quality. Their response times were recorded using E-Prime 3.0. The trials were distributed across four counterbalanced lists based on a Latin square design to control for potential order effects. The process is shown in Figure 1.

In the second phase, participants took part in post-task interviews designed to elicit their explicit reasoning. These interviews involved open-ended questions, such as “Did line order affect your ratings?”, “Did differences in expression within a line influence your judgment of translation quality?”, and “What aspects did you consider when making your judgment?” Participants’ responses were audio-recorded and later coded in binary form (Yes/No) to facilitate subsequent analysis. This binary coding simplifies nuanced responses and is acknowledged as a limitation.

### 4.5. Data Analysis

All data were analyzed using SPSS 26.0 (IBM Corp., Armonk, NY, USA) following a series of predefined steps.

First, translation quality ratings were normalized by converting the original 1–5 star scores to a continuous 0–1 scale, with each increment corresponding to 0.2. Descriptive statistics, including mean ratings and response times (RTs), were then computed for each experimental condition.

Given the known properties of RT data, several preprocessing steps were applied. No extremely fast responses (e.g., <300 ms), which are typically considered anticipatory, were observed in the dataset. RTs exceeding ±2.5 standard deviations from each participant’s mean were trimmed to reduce the influence of outliers.

This trimming procedure also reduced the influence of extremely long response times observed in the raw data. No logarithmic transformation was applied. After trimming, visual inspection of the RT distribution suggested no substantial skewness or extreme values that would necessitate additional transformation. Raw RTs were retained to preserve interpretability.

To examine the effects of phrase order and local variation in semantic expression, separate two-way repeated-measures analyses of variance (ANOVAs) were conducted on rating scores and RTs, testing for main effects and interactions between the two factors. Where appropriate, post hoc comparisons were performed using paired-sample *t*-tests with Bonferroni correction.

To assess the potential influence of item-level variability, additional item-level inspections were conducted. Mean responses were examined across items to evaluate the consistency of condition effects. The direction of effects was generally consistent across items, with no indication that the overall pattern was driven by a small subset of stimuli.

Although mixed-effects models are often recommended for simultaneously accounting for participant- and item-level variability, the present study adopted repeated-measures ANOVA due to the balanced design and controlled stimulus construction. Nevertheless, the supplementary item-level checks provide supporting evidence that the observed effects are robust across the stimulus set.

For the analysis of verbal reports, participants’ responses to post-task interview questions were coded into binary categories (e.g., “Yes” vs. “No”) to indicate the presence or absence of explicit awareness of specific experimental manipulations. A one-sample *t*-test was conducted against the chance level of 0.5 to assess whether participants’ explicit awareness differed from random responding. This binary coding scheme necessarily simplifies nuanced verbal data and is acknowledged as a methodological limitation.

Effect sizes were reported alongside significance tests, with Cohen’s *d* for *t*-tests and partial eta-squared (ηp^2^) for ANOVA. The significance threshold was set at α = 0.05 for all inferential analyses.

## 5. Results

The results are presented in two main parts. Section 5.1 reports the quantitative findings from the behavioral task, detailing the effects of phrase order and semantic expression on quality ratings and response times. Section 5.2 summarizes the qualitative and binary responses elicited from the post-task verbal reports.

### 5.1. Behavioral Data (Phase One)

A 2 (Phrase Order: congruent vs. incongruent) × 2 (Semantic Expression Type: literal vs. non-literal) repeated measures ANOVA was conducted on the quality ratings. Both by-subject (F_1_) and by-item (F_2_) analyses were performed.

**Figure 2:**
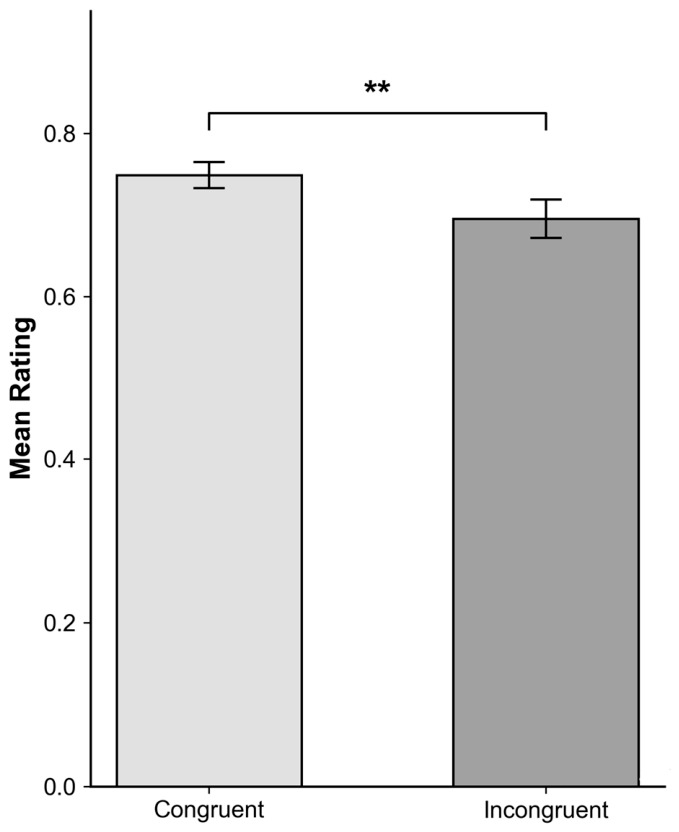
Mean translation quality ratings (±SE) by Phrase Order condition. ** indicates *p* < 0.05.

The analysis revealed a significant main effect of Phrase Order, F_1_(1,43) = 6.17, *p* = 0.004, ηp^2^ = 0.18; F_2_(1,8) = 13.46, *p* = 0.006 in rating. Specifically, translations with congruent phrase order (M = 0.75, SD = 0.11) were rated significantly higher than those with incongruent order (M = 0.696, SD = 0.16), *t*(43) = 2.720, *p* = 0.009, *d* = 0.35 (Figure 2). This indicates that participants generally rated the congruent phrase order differently than the incongruent order.

**Figure 3:**
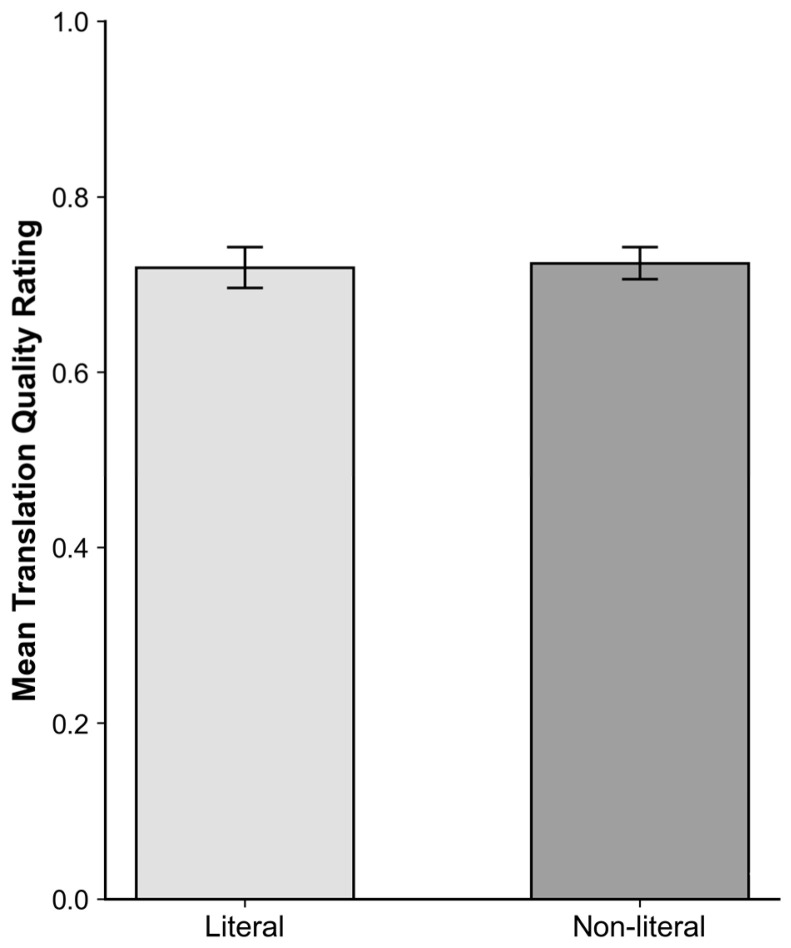
Mean translation quality ratings (±SE) by Semantic Expression Type condition.

The main effect of Semantic Expression Type was not significant, both conditions yielded similar average scores around 0.72 (Figure 3); F_2_(1,8) = 0.016, *p* = 0.902.

**Figure 4:**
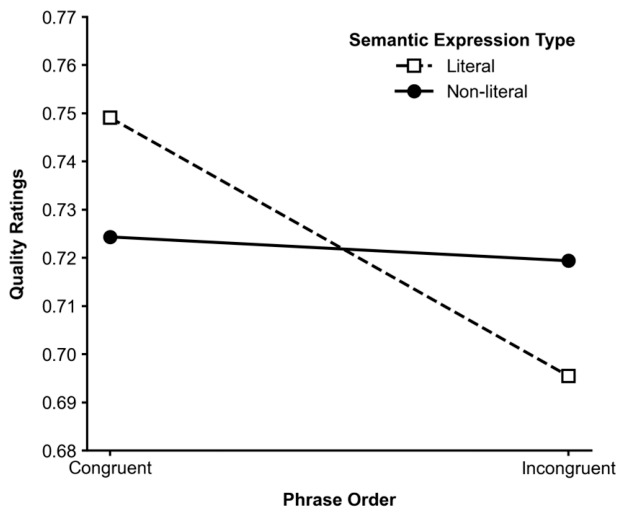
Interaction between Phrase Order and Semantic Expression Type in quality rating.

Importantly, a significant interaction between Phrase Order and Semantic Expression Type was found for quality ratings, F_1_(1,43) = 6.17, *p* = 0.017, ηp^2^ = 0.125; F_2_(1,8) = 8.50, *p* = 0.019. As illustrated in Figure 4, there was a clear interaction between semantic expression type and phrase order on quality ratings. Specifically, when the phrase order was congruent with the English reference, the literal expression received the highest ratings (M = 0.749). However, when the phrase order was incongruent, the preference for literal translation reversed, with its rating dropping to the lowest across all conditions (M = 0.695). The ratings for non-literal expressions remained relatively stable across the congruent (M = 0.724) and incongruent (M = 0.719) conditions.

**Figure 5:**
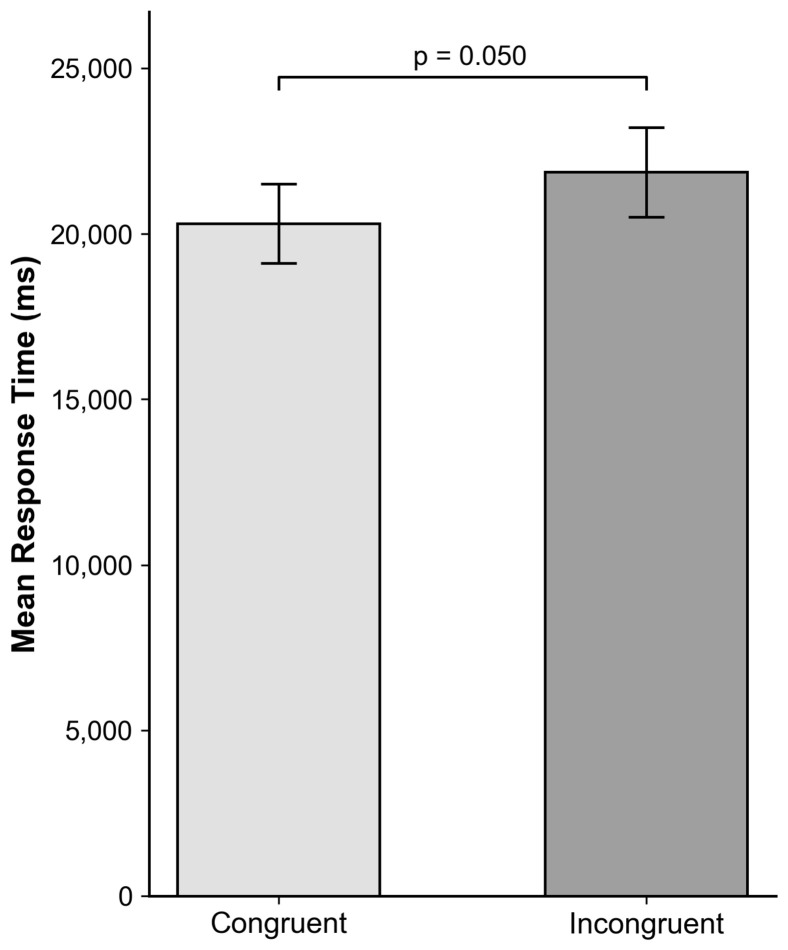
Mean response time (ms) (±SE) by Phrase Order Condition.

A similar 2 × 2 repeated measures ANOVA was conducted on the response times. The results yielded a marginal main effect of Phrase Order in the item analysis, F_1_(1,43) = 3.723, *p* = 0.06, ηp^2^ = 0.08; F_2_(1,8) = 5.58, *p* = 0.046. Participants tended to evaluate congruent translation (M = 20,314.05 ms, SD = 7904.13) faster than the incongruent order condition (*M* = 21,868.57 ms, *SD* = 8994.09), *t*(43) = 2.012, *p* = 0.050, *d* = 0.30 (Figure 5). Suggesting that structural alignment significantly influenced the processing speed.

**Figure 6:**
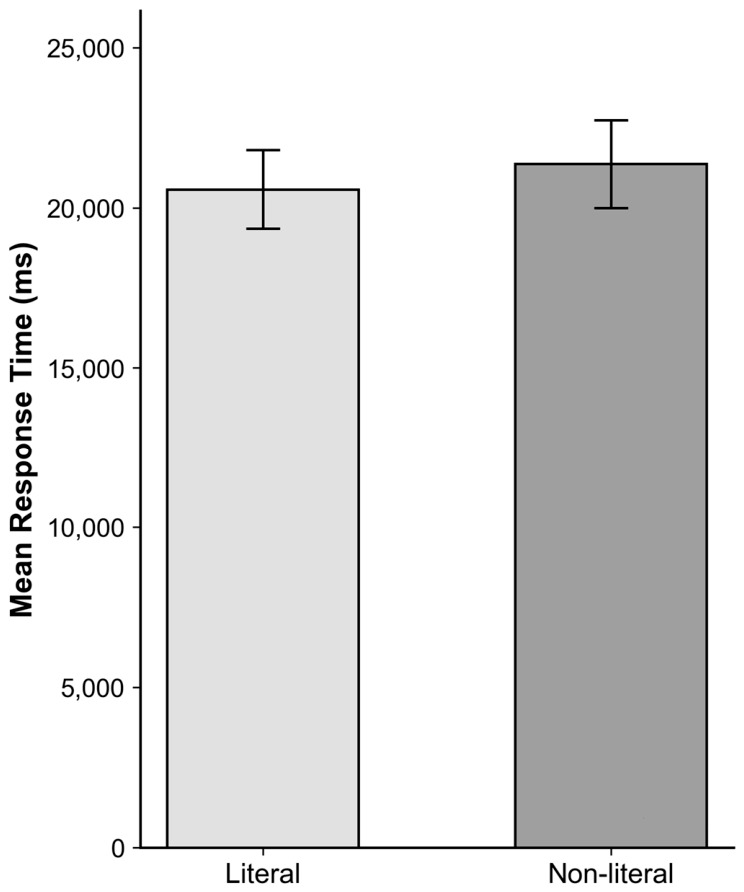
Mean response time (ms) (±SE) by Semantic Expression Type condition.

There was no significant main effect of semantic expression type, though descriptive data showed that items with non-literal expressions (M = 21,371.31 ms) were judged slightly slower than those with literal expressions (M = 20,579.46 ms) (Figure 6).

**Figure 7:**
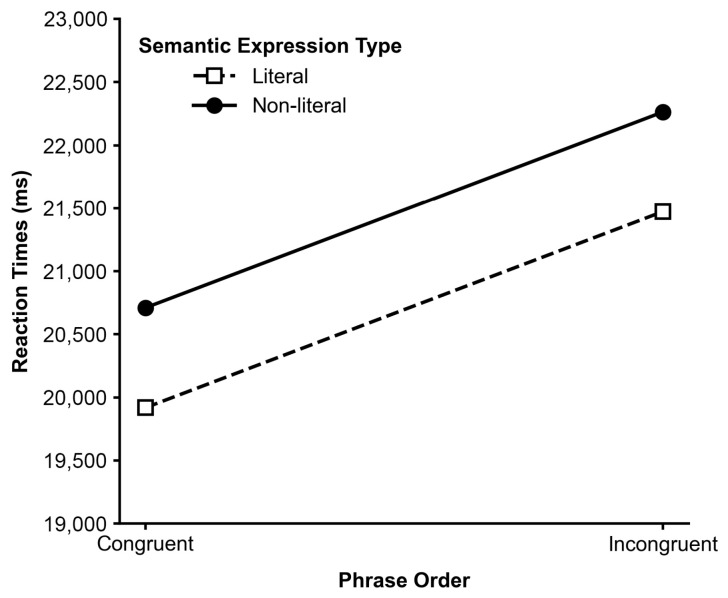
Interaction between Phrase Order and Semantic Expression Type in response time (ms).

There was no significant main effect of Semantic Expression Type on reaction times, F_1_(1,43) = 0.469, *p* > 0.05; F_2_(1,8) = 0.099, *p* = 0.761. Furthermore, the interaction between Phrase Order and Semantic Expression Type was not significant, F_1_(1,43) = 0.092; F_2_(1,8) < 0.001, *p* = 0.989. This lack of interaction (Figure 7) implies that the additional processing cost incurred by evaluating structurally incongruent items was consistent regardless of whether the semantic expression was literal or non-literal.

### 5.2. Verbal Report (Phase Two)

When asked, “*Did the reversed line order in the target text affect your quality judgment, for example, lower your rating?*”, results showed that 72.7% of participants (*n* = 32/44) explicitly denied any influence of phrase order on their evaluations.

For the question “*Did differences in expression within a line influence your judgment of translation quality?*”, 80% of participants initially provided ambiguous answers (e.g., “*It depends*”). When pressed to give a definitive Yes/No response, answers split nearly evenly: 21 participants (47.7%) affirmed an influence, while 23 (52.3%) denied it.

## 6. Discussion

This study aimed to uncover the cognitive dynamics underlying translation evaluation by comparing behavioral metrics with explicit verbal reports. This section first discusses participants’ distinct responses to phrase order manipulation (Section 6.1) and variation in semantic expression (Section 6.2). It then explores the significant interaction between these two linguistic variables (Section 6.3). Finally, it theorizes the observed divergence between behavioral and verbal data through the lens of dual-system theories and processing fluency (Section 6.4).

### 6.1. Behavioral and Verbal Responses to Phrase Order Manipulation

The present findings suggest that behavioral indicators and verbal reports capture different aspects of participants’ responses to phrase order manipulation. Behavioral data, including response times and rating patterns, appear to be more directly influenced by processing demands, whereas verbal reports reflect participants’ explicit interpretations of their decision-making processes. Rather than indicating a strict dissociation, this divergence may arise from differences between implicit processing and explicit reflection. More specifically, the three measures showed different levels of sensitivity to phrase order manipulation. Response times reflected increased processing effort under incongruent conditions, rating scores showed a weaker but consistent evaluative effect, whereas verbal reports largely failed to capture this influence, indicating limited explicit awareness of phrase order as a factor in evaluation.

#### 6.1.1. Processing Effort and Structural Fluency

One plausible explanation for the observed pattern lies in differences in processing effort. When phrase order deviates from expected structures, participants are likely to require additional cognitive resources to align source and target meanings. This increased effort is reflected in longer response times (Δ = 1554 ms) and lower ratings (Δ = 0.35) for incongruent-order translations (Figure 2 and Figure 5). In contrast, congruent structures facilitate more efficient processing, leading to faster responses and higher perceived quality.

From this perspective, participants’ preference for congruent phrase order may be driven by processing fluency rather than an explicit evaluation of translation quality per se. This interpretation is consistent with psycholinguistic research showing that structurally predictable input reduces cognitive load and enhances perceived acceptability.

At the same time, verbal reports did not reliably reflect this influence: 72.7% of participants explicitly denied that phrase order affected their judgments. This discrepancy can be understood in terms of the difference between online processing and retrospective reporting. While processing difficulty is experienced during task performance, it may not be readily accessible to conscious awareness or may not be considered a legitimate basis for evaluation when participants articulate their reasoning.

#### 6.1.2. Implicit Sensitivity and Conflict Detection

The observed divergence between behavioral and verbal data may also be interpreted in terms of implicit sensitivity to structural variation. Incongruent phrase orders are likely to create a mismatch between participants’ syntactic expectations and the presented structure, requiring additional effort to resolve. Although participants did not explicitly report sensitivity to such discrepancies, increased response times suggest that these mismatches were detected and processed at an implicit level.

This interpretation is compatible with accounts of conflict detection in cognitive processing, according to which the system continuously monitors for inconsistencies and allocates additional resources when discrepancies arise. In the present study, the increased cognitive effort associated with resolving phrase order differences may have influenced both response times and, to a lesser extent, evaluation outcomes, even in the absence of explicit awareness.

Importantly, this does not necessarily imply that participants consciously evaluated syntactic features. Rather, structural variation may have affected the ease with which meaning was processed, which in turn shaped evaluative responses.

#### 6.1.3. Methodological Implications

These findings have several methodological implications. Behavioral measures, such as response times, provide access to online processing dynamics and can capture variations in cognitive effort that may not be reflected in self-reported data. In contrast, verbal reports are retrospective in nature and depend on participants’ ability and willingness to articulate the factors underlying their judgments.

It should also be noted that participants in the present study were bilinguals rather than trained translators. Their evaluations are therefore likely to reflect subjective perceptions of fluency and ease of processing, rather than objective assessments of translation quality. From this perspective, the observed effects may be more appropriately interpreted as reflecting target text perception rather than strictly translation quality evaluation.

Verbal reports may also be influenced by social desirability bias, by general beliefs about translation (e.g., preferences for meaning over form) or by the limitations of binary questioning formats. When asked to provide explicit explanations to justify their judgments, participants sometimes appeared to rely on general beliefs about translation (e.g., “literal translation is rigid”) rather than on the transient cognitive processes that guided their decisions during task performance.

Taken together, the results suggest that behavioral indicators and verbal judgments should be viewed as complementary rather than competing sources of evidence. Behavioral data capture aspects of processing that operate below the level of conscious awareness, while verbal reports provide insight into how participants interpret and rationalize their judgments.

### 6.2. Effects of Semantic Expression Type on Processing and Evaluation

The results suggest that variation in semantic expression type influenced processing speed but had a limited impact on explicit evaluation. While rating scores did not differ reliably across conditions (both conditions: M = 0.72; Figure 3), response times were longer for non-literal expressions (Δ = 792 ms; Figure 6), indicating increased processing effort.

Rather than reflecting a strong dissociation, this pattern may be more appropriately interpreted in terms of differences in sensitivity across measures. Response times appear to capture subtle variations in processing difficulty, whereas rating scores reflect more global evaluative judgments that may not be sensitive to localized differences in expression.

#### 6.2.1. Processing Effort and Variation in Expression

One plausible explanation for this pattern is that non-literal expressions introduce additional processing demands. When meaning is realized through alternative phrasing rather than closely aligned formulations, participants may need to engage in additional interpretive effort to establish equivalence between source and target texts. This increased effort is reflected in longer response times.

In contrast, explicit ratings remained largely unaffected. This may be because participants relied on overall meaning and coherence rather than on fine-grained differences in expression when making evaluative judgments. As long as the intended meaning could be successfully inferred, variation in expression may not have been considered a critical factor in explicit evaluation.

The absence of an effect on explicit ratings may also be related to the localized nature of the manipulation. Because variation in semantic expression was restricted to individual components and did not substantially alter global meaning, it may not have been sufficiently salient to influence overall evaluative judgments. This is consistent with psycholinguistic accounts suggesting that local variations that do not disrupt global meaning are less likely to influence holistic evaluative judgments, even when they increase processing effort.

This interpretation suggests that differences in semantic expression operate at a level that influences processing effort without necessarily reaching the threshold required for conscious evaluation, particularly for non-expert participants.

#### 6.2.2. Implications for Measurement Sensitivity

These findings highlight differences in the sensitivity of behavioral and evaluative measures. Response times provide an index of online processing effort and can reveal the cognitive cost associated with interpreting alternative expressions. In contrast, rating scores require participants to integrate multiple evaluative dimensions, including fluency, coherence, and perceived adequacy, which may reduce sensitivity to variation within individual components.

It should also be noted that participants in the present study were bilinguals rather than trained translators. Their evaluations are therefore likely to reflect overall interpretability rather than detailed sensitivity to how meaning is expressed. From this perspective, the absence of rating differences does not necessarily indicate that variation in expression is irrelevant, but rather that it may not play a prominent role in explicit evaluation under the present task conditions.

Taken together, the results suggest that local variation in semantic expression within individual components (e.g., within a single line) primarily affects processing effort rather than overt evaluative outcomes. This further supports the view that behavioral and explicit measures capture different aspects of translation processing, and should be interpreted as complementary sources of evidence.

### 6.3. Interaction Between Phrase Order and Local Semantic Expression: Implications for Resource Allocation

A particularly informative finding is the significant interaction between phrase order and semantic expression type observed in rating data (Figure 4), which was not present in response time measures (Figure 6). This pattern suggests that the influence of local semantic variation may depend on the structural configuration of the text during evaluative processing.

One possible explanation is that structural processing demands associated with incongruent phrase order may reduce the extent to which participants attend to fine-grained semantic differences. When phrase order is congruent, structural processing is relatively efficient, which may allow greater sensitivity to local semantic variation, reflected in clearer differences between literal and non-literal expressions in ratings. In contrast, when phrase order is incongruent, increased processing demands at the structural level may reduce the salience of semantic distinctions during global evaluation.

The absence of a corresponding interaction in response times suggests that this modulation does not emerge at the level of online processing effort. Instead, it may reflect how different sources of linguistic information are integrated during the formation of explicit evaluative judgments, with structural processing exerting a stronger constraint under higher cognitive demand conditions.

### 6.4. Dissociation Between Conscious Awareness and Automatic Processes in Translation Evaluation

The present findings suggest a divergence between conscious awareness (as reflected in verbal reports) and automatic cognitive processes (as reflected in behavioral measures) during translation evaluation. Rather than indicating a strict dissociation, this pattern points to differences in the level of processing captured by different measurement modalities.

In translation evaluation, phrase order manipulation—a syntactic-pragmatic feature operating at the structural level—was processed rapidly and implicitly, consistent with automatic System 1 processing, whereas verbal reports required deliberate reflection and causal justification, consistent with System 2 reasoning. Behavioral data indicated that congruent phrase order was associated with higher ratings (*d* = 0.35) and marginally faster response times (*d* = 0.30), suggesting reduced processing effort under structurally aligned conditions. However, when asked explicitly about the influence of phrase order, 72.7% of participants denied its impact, indicating limited metacognitive access to this effect. This pattern suggests that automatic processing may influence evaluative behavior without necessarily being accessible to conscious explanation.

This divergence becomes evident when considering the interaction between phrase order and semantic expression type. A significant interaction was observed, whereby the effect of semantic expression type on ratings was more pronounced under congruent phrase order than under incongruent order. This conditional effect is consistent with dual-system accounts. In structurally coherent contexts (congruent order), reduced processing demands may allow participants to allocate greater attention to local semantic variation within individual components (i.e., within-line expression differences), thereby increasing sensitivity to semantic expression type. In contrast, when phrase order is incongruent, increased structural processing demands may reduce available cognitive resources for evaluating local semantic variation, thereby attenuating its influence on explicit judgments.

This pattern suggests an adaptive allocation of cognitive resources during evaluation, in which structural processing constraints modulate sensitivity to local semantic variation. Importantly, semantic expression variation in this study was localized within individual lines and did not alter the global meaning of the stimulus, which may further explain why its effects were not consistently reflected in explicit ratings.

#### 6.4.1. Attribution Errors and Fluency Heuristics

Participants likely misattributed processing fluency arising from structural alignment to general judgments of translation quality. According to the processing fluency theory (Reber et al., 2004), fluency is typically experienced as a positive cognitive state, but its underlying sources are not always accurately identified during conscious reflection.

Within this framework, structural alignment between source and target texts may facilitate more efficient online processing, generating a subjective sense of cognitive ease. This ease may subsequently contribute to higher quality ratings for congruent phrase order conditions, even though participants are not explicitly aware of the structural basis of this effect.

From a dual-system perspective, such fluency effects can be understood as emerging from rapid, automatic processing routines (System 1), whereas verbal reports rely on slower, reflective reasoning processes (System 2). However, the extent to which System 2 accurately accesses the sources of these processing experiences remains limited, particularly in retrospective interview settings.

This interpretation is consistent with the present findings, where participants’ verbal explanations frequently referred to general notions such as “flow” or wording-based criteria, rather than specific structural properties of the stimuli. This suggests that evaluative judgments may be shaped by processing-based experiences that are not fully represented in explicit reasoning.

#### 6.4.2. Social Desirability and Normative Influences on Verbal Reports

Verbal reports may also be influenced by normative beliefs and socially shared assumptions about translation rather than direct access to online cognitive processes. In post-task interviews, participants frequently referred to general translation principles such as “literal translation is rigid,” reflecting widely held evaluative norms rather than task-specific processing behavior.

Such responses may reflect the tendency for participants to rely on culturally or educationally acquired beliefs when articulating their judgments. This tendency may be particularly evident in bilingual participants, whose explicit reasoning about translation may be shaped by general language ideologies rather than moment-to-moment processing dynamics.

When asked about the influence of semantic expression type (i.e., local variation within individual lines), many participants responded with general or non-committal answers such as “it depends,” suggesting that such fine-grained variations were not consistently accessible at the level of explicit reflection. This does not imply absence of sensitivity at the processing level, but rather a potential dissociation between implicit processing and explicit articulation.

#### 6.4.3. Temporal Decoupling: Limited Access to Online Processing Cues

Another factor that may contribute to the observed dissociation between behavioral responses and verbal reports is the temporal gap between online processing and retrospective reporting. Behavioral responses, including response times and ratings, reflect moment-to-moment processing during task performance, whereas verbal reports are generated after task completion and rely on reconstructed accounts of the decision process.

Given this temporal separation, participants may have limited access to the fine-grained processing dynamics that occurred during evaluation, such as brief increases in processing effort when encountering incongruent phrase orders or locally non-literal semantic expressions within individual lines. Instead, post-task explanations are more likely to be based on generalized evaluative beliefs (e.g., preferences for meaning over form) rather than transient processing states.

From this perspective, the absence of explicit awareness of specific manipulation effects does not necessarily indicate a lack of sensitivity at the processing level, but may reflect constraints on introspective access to rapid, localized cognitive operations.

## 7. Conclusions

This study investigated differences between behavioral indicators (response times and rating scores) and verbal reports in translation quality evaluation, focusing on phrase order and semantic expression type.

The results show a consistent pattern across both manipulations. Phrase order significantly affected behavioral responses, with congruent structures associated with faster response times and higher ratings, whereas most participants did not report being influenced by phrase order. Local semantic expression type affected response times but had no significant effect on explicit ratings.

Overall, behavioral measures were more sensitive to the perception of experimental manipulations in translation evaluation than verbal reports, indicating that these measures capture different aspects of translation evaluation.

These findings contribute to a better understanding of translation evaluation as a process involving both automatic and controlled components, highlighting the value of implicit behavioral measures in capturing processing effects not fully accessible through verbal reports. The results also have implications for translation training, particularly in raising learners’ awareness of how structural and lexical factors influence evaluative decisions.

The study is limited by the use of non-expert bilingual participants and short poetic stimuli, which may constrain generalizability. Future research may further explore these effects using professional translators, additional text types, and more advanced analytical methods.

## Figures and Tables

**Table 1:** Representative examples of the four experimental conditions.

Condition	Source Text (Chinese)	Target Text (English)
Congruent + Literal	黑暗电视	dark television
	娱乐亡灵	entertains lost spirits
	空房间	of an empty room
Incongruent + Literal	空房间	dark television
	黑暗电视	entertains lost spirits
	娱乐亡灵	of an empty room
Congruent + Non-literal	黑白电视	dark television
	娱乐亡灵	entertains lost spirits
	空房间	of an empty room
Incongruent + Non-literal	空房间	dark television
	黑白电视	entertains lost spirits
	娱乐亡灵	of an empty room

## Data Availability

The data supporting the findings of this study are openly available in Harvard Dataverse (https://doi.org/10.7910/DVN/PG4QGK). The dataset, titled Experiment Materials: Chinese-English haiku pairs selected from Lighting the Bridge to the Moon: One Hundred and Eleven Macao Haiku, contains the experimental stimuli used in this study.
